# An Electrical Resistivity Method of Characterizing Hydromechanical and Structural Properties of Compacted Loess During Constant Rate of Strain Compression

**DOI:** 10.3390/s20174783

**Published:** 2020-08-25

**Authors:** Pengju Qin, Yubo Liu, Zhiwei Song, Fuli Ma, Yongbao Wang, Xiao Zhang, Chenxi Miao, Xiaoqiang Dong

**Affiliations:** 1 College of Civil Engineering, Taiyuan University of Technology, Taiyuan 030024, China; Yuboliu20@163.com (Y.L.); geiliyoubao@163.com (Z.S.); mafuli@tyut.edu.cn (F.M.); yonbaowang@163.com (Y.W.); zhangxiao01@tyut.edu.cn (X.Z.); miaochenxi@tyut.edu.cn (C.M.); dongxiaoqiang@126.com (X.D.); 2 Shanxi Transportation Technology Research and Development Co. Ltd., Taiyuan 030032, China; 3 College of Mining Engineering, Taiyuan University of Technology, Taiyuan 030024, China

**Keywords:** compacted loess, electrical resistivity, constant rate of strain, moisture content, hydromechanical and structural properties

## Abstract

Hydromechanical and structural properties of compacted loess have a significant impact on the stability and reliability of subbase and subgrade, which needs to be quickly determined in the field and laboratory. Hence, an electrical resistivity method was used to characterize the hydromechanical and structural properties of compacted loess during constant rate of strain compression. In the present work, compacted loess samples with a dry density of 1.7 g/cm^3^, a diameter of 64 mm, a height of 10 mm and different water content ranging from 5–25% were prepared. The constant rate of strain (CRS) tests were conducted by a developed oedometer cell equipped with a pair of horizontal circular electrodes (diameter of 20 mm) and vertical rectangular electrodes (width of 3.5 mm) to determine the electrical resistivity of compacted loess. The results showed that as average water content increases, plastic compression indices increase from 0.220 to 0.350 and the elastic compression indices increase from 0.0152 to 0.030, but they decrease to 0.167 and 0.010 and yield stress decreases from 381.28 kPa to 72.35 kPa. Moreover, as vertical strain increases, the variation trend of average formation factor and average shape factor for the lower water content decreases but increases for the maximum water content, and the anisotropy index first decrease and then tend to increase slightly, which indicates that the structural properties of unsaturated and saturated samples during compression exhibits different trend and the anisotropy of samples tend to be stable as vertical strain increases. As the water content increases, the average formation factor and average shape factor decrease, but the anisotropy index first decreases then increases, suggesting that water content has a significant impact on these electrical indices. More important, The coefficients of average formation factor decrease from 33.830 to −1.698 and the coefficients of average shape factor decrease from 8.339 to −0.398 as water content increases, whereas there is less variation for the coefficient of anisotropic index with a value of 2.190. An equation correlating average formation factor and water content and vertical strain is regressed to characterize the hydromechanical properties of compacted loess by measuring its impedance, which can be used to evaluate the stability of compacted loessic ground and subgrade.

## 1. Introduction

As the ‘the great western development strategy’ and ‘mid-to-long term railway network plan’ in 2001 and 2008 were approved, respectively, the numbers of building and length of high-speed railways and highways in China have experienced a rapid growth. During the construction, loess are always used as the material of subbase and subgrade in the loess area. It is acknowledged that the Loess Plateau in northern and northwestern China occupies more than 6% of the territory in China [1]. Loess is a yellowish, open-structured, silty, aeolian deposit, which is also widely distributed over the world [2,3]. However, China has the most complete topographic feature of loess (i.e., an total area of 6.3 × 10^5^ km^2^) and the thickest loess deposit in the world [1,4,5]. In the engineering practice, loessic soil is initially compacted to improve the stability of the ground and subgrade, which is often subjected to further loading for constructing buildings or roads. The hydromechanical and structural properties of compacted loess are sufficiently significant to influence the stability and reliability of the loess as ground and subgrade. Therefore, how to quickly determine the hydromechanical and structural properties of the soil is significant to evaluate safety and stability of a practical project.

Compared to traditional incremental loading tests, the constant rate of strain (CRS) consolidation test is a standardized, efficient and relatively rapid method to determine consolidation properties. Moreover, CRS tests can obtain continuous data—rather than discrete data based on load increment size—which can improve the accuracy of consolidation mechanical parameters [6]. CRS tests are used to investigate compressibility of unsaturated soils in the recent decades [7,8,9,10,11,12]. In [9], a static compaction apparatus was developed to study the post-compaction suction change of a kaolin clay by applying a loading rate of five kilopascals per minute under a constant water content condition. Muñoz-Castelblanco et al. [13] investigated the compression and collapse behavior of a natural unsaturated loess by running a series of constant rate of strain (within 0.003–0.059% strain per min) compression tests. In addition, a rate of two micrometers per minute was adopted for studying the compression properties of unsaturated silt soil [14]. Qin et al. [12] studied influence of strain-rate on hydromechanical behavior of highly compacted bentonite by single-stage and stepwise CRS tests at ambient temperature and verified the applicability of the isotache concept on the compacted bentonite.

Electrical resistivity can be used to study the structural properties of soil. Electrical resistivity is a basic parameter to describe the conductivity of soil and closely linked to other soil physical and mechanical parameters. Archie [15] first studied the relationship between resistivity measurements and formation characteristics and established the relationship model between the formation resistivity factor and porosity. It have presented that the formation factor depends on porosity, degree of saturation, particle orientation and particle shape, contact orientation and cementation [16,17]. Dafalias and Arulanandan [18] derived the average formation factor and average shape factor that take into account the orientation of particles with an assumption of transverse isotropy. It shows that the average formation factor and average shape factor are independent of anisotropy caused by preferred particles orientation. The average shape factor is a function of shape of the particles and cementation [19]. Arulanandan and Kutter [20] proposed anisotropy index A to quantify the anisotropy of particles. The primary model is suitable for saturated cohesionless soil and pure sand and extended to include particle surface conduction and to describe the electrical resistivity of various types of soil at saturated and unsaturated states [21,22,23]. Soil resistivity can reflect the composition and structure characteristics of soil [24]. The resistivity method can be used to quantitatively evaluate the microstructure deformation characteristics of soil, measure water content, analyze the composition of soil particles and determine the engineering mechanical properties of soil [25,26,27,28,29].

The present work aims to investigate an electrical resistivity method of characterizing the hydromechanical and structural properties of compacted loess samples under CRS loading. Cylindrical samples with different water content of 5–25% were prepared by using loess powder that had been sprayed with water. Then, the samples were subjected to CRS compression; the vertical and horizontal alternating current (AC) impedance of the samples was measured during compression. Based on the experimental results, compression deformation curves were obtained, and mechanical parameters were computed. Moreover, the electrical parameters that are behalf of the microstructure of soil are determined and the relationships between water content and vertical strain and the electrical parameters were analyzed. A equation is proposed to characterize the hydromechanical properties of compacted loess. This work can provide a basis for evaluating safety and stability of compacted loess soil.

## 2. Materials and Methods

### 2.1. Material

**Figure 1:**
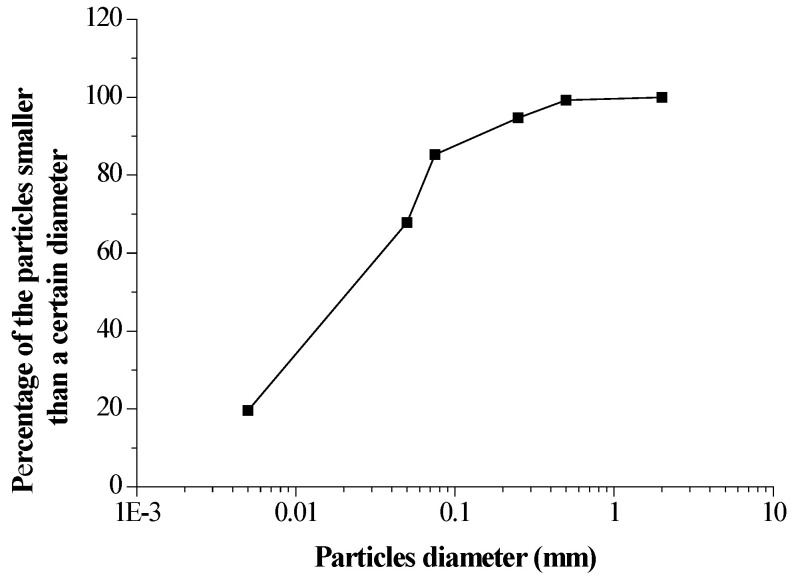
Grain-size distribution curve.

The loess used in this study was extracted from an expressway site in Xinzhou City, Shanxi Province. The intact soil is relatively uniform, yellow and stiff. Table 1 presents the basic physical parameters of the soil. The particle distribution curve is shown in Figure 1. According to the standard for engineering classification of soil [30], the loess used in this study can be classified into silt because the content of fine particles is more than 50% and the plasticity index I_P_ < 10. The XRD analysis shows fraction of the detected five minerals: quartz (57%), albite (18%), calcite (13%), muscovite (6%) and chlorite (6%). Quartz, albite, muscovite and calcite are the non-clay or silt minerals and chlorite is the clay minerals.

For preparation of the specimens, loess powder with a hygroscopic water content of 1.2% was mixed with water to prepare loess powder with a given water content. In the present study, water contents were selected as 5%, 10%, 15%, 20% and 25%. To prepare the soil powders with a designated water content, a predetermined volume of water was sprayed to a predetermined amount of the air-dried soil that passed through 2-mm net sieves. Then, they were thoroughly mixed for 5 min to achieve preliminary homogeneity, followed by enclosing the soil in a plastic bag for 24 h to make water evenly distribute in the soil.

Sequentially, based on a target dry density of 1.7 g/cm^3^ and a diameter of 64 mm and a height of 10 mm, the loess powder with a given mass was put into the sample ring and stirred evenly by a fine bar. A metal cylinder was then used to statically compact the powder with a constant rate of displacement of 0.4 mm/min, which was loaded by a loading frame. After compressing the soil to the designed height, the shape of the sample was held unchanged for an hour to reduce rebound during unloading and enhance the homogenization of the sample. After compaction, the sample was dismantled from the loading frame and its initial height was measured by a digital caliper. The process above was repeated until all of the samples were manufactured; the specifications of as-compacted loess samples are shown in Table 2.

### 2.2. Apparatus and Calibration

In this section, the setup used is introduced and an according calibration was performed to provide a basis for eliminating a systemic error of tests.

**Figure 2:**
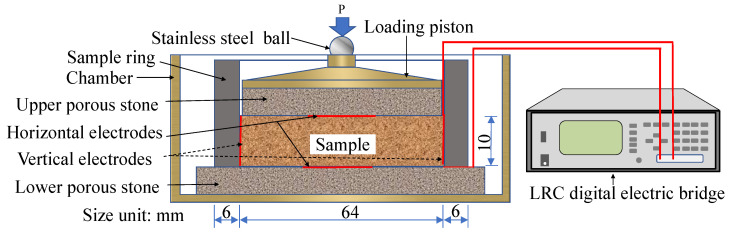
Setup of an oedometer cell with measurement of sample impedance.

Figure 2 is the setup of an oedometer cell with measurement of sample impedance. The oedometer cell used in this work was developed to implement CRS tests on compacted loess samples and mainly consists of a chamber, a sample ring, a loading piston, a stainless steel ball, electrodes and two pieces of porous disks. The sample had an initial height of 10 mm. The sample ring was made of high-strength and rigid insulating material and had an inner diameter of 64 mm. A pair of horizontal circular electrodes made of copper foil was set at the top and bottom surface of the sample, while a pair of vertical rectangle copper electrodes was symmetrically and radially installed at two lateral sided of the sample. The horizontal electrodes had a diameter of 20 mm and the vertical electrodes had a width of 3.5 mm, both of the electrodes had a thickness of 0.05 mm. The horizontal and vertical electrodes were alternately connected with the LRC digital electric bridge to measure the vertical and horizontal electrical impendence of samples, respectively. In order to conveniently connect electrode with metal clip linked with the LRC digital electric bridge, one end of fine electric wire was adhered to the center of the electrodes and the other end stretched out the oedometer cell to link with the metal clip. The sample, two pieces of filter paper and horizontal electrodes were sandwiched between the upper and lower porous stones. The upper porous stone was placed in the sample ring, whereas the lower porous stone supports the sample ring, electrodes and sample and its size was larger than the outer diameter of the sample ring. In addition, the filter papers were placed close to the porous stones and not drawn in Figure 2. A metal loading piston was over the upper porous stone to bear the load imposed by a loading frame and delivers the loading to the sample. In order to eliminate impact of the metal piston on impedance measurement of the sample, the porous stones were initially desiccative and fine electric wires to connect electrodes with LRC digital electric bridge were packaged with insulated skin. A stainless-steel ball was placed on the loading piston to made the loading evenly impose on the loading piston. The sample ring, porous stone and so forth were placed into a metal chamber.

In this work, the LCR digital bridge TH2828A was adopted to measure the electrical impedance of samples. The instrument had an accuracy of 0.1% and a frequency range of 20 Hz–1 MHz and an AC frequency range of 50 Hz–1 MHz was used in the study. The effective working range of the instrument was 0.1 Ω to 100 MΩ. The sensing technique for the unknown impedance was to balance the digital AC bridge through freely controlling the amplitude and phase shift of voltage source linked with the unknown impedance. Consequently, the unknown impedance could be computed from the reference impedance, reference voltage source and controlled voltage source and the details could refer to [31]. Due to measurement performed on both ends of the sample, a two-point measurement was used herein and resembles with that of the two-electrode soil box outlined in [32]. In this study, the precision digital LCR meter was used to measure the module of impedance |Z| of compacted loess samples and the resistivity values were calculated by Equation (1):

ρ = |Z|S/L(1)

where, ρ is the loess resistivity (Ω·m); |Z| is the impedance mode (Ω); S is the electrode area (m^2^); L is the distance between the electrodes (m).

**Figure 3:**
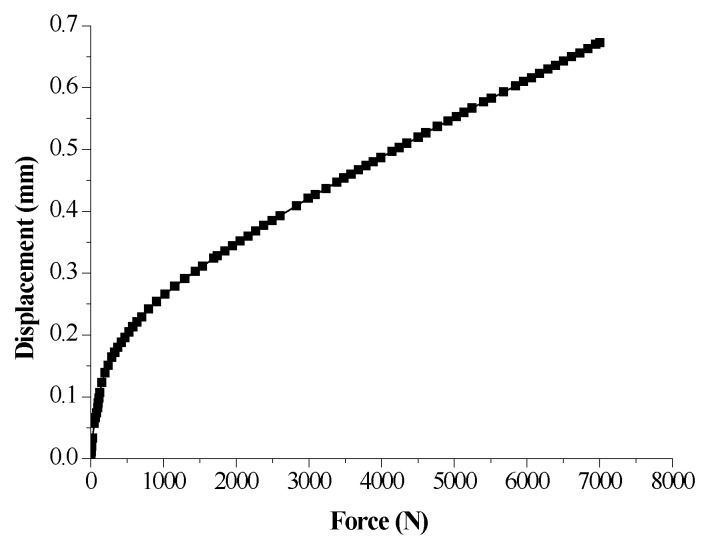
Calibration curve of the oedometer cell.

For the sake of eliminating impact of setup deformation on real deformation of the sample during compression, the calibration curve of the oedometer cell as shown in Figure 3 was obtained by taking the place of the sample with a metal cylinder. An initial force of 1 kPa was applied to tighten contact of all components of the oedometer cell and a displacement rate that is the same with CRS tests of samples was adopted to compress the oedometer cell and obtain the curve of force and displacement. The calibration data need to be subtracted from the CRS test data of samples.

### 2.3. Test Procedures

After compaction, the sample was installed into the oedometer cell chamber with the sample ring. Prior to the sample placement, the lower horizontal electrodes, filter paper and porous stone under the sample was installed in the chamber. Note that both of the vertical electrodes had been installed into the inner side of the sample ring before preparation of the sample. After placement of the sample, the upper horizontal electrodes, filter paper, porous stone, loading piston and stainless steel ball over the sample was installed in the chamber. Then, the electrodes were connected with the LCR digital electric bridge with fine electric wires.

After sample installation, an initially vertical pressure of 1 kPa was imposed on the sample to facilitate the loading piston and the electrodes better contact with the sample. During the CRS tests, a strain rate of 1.00 × 10^−5^ s^−1^ was adopted after referring to many previous works [33,34,35] to obtain the compression curves of the compacted loess sample on an ambient temperature. In addition, the displacement was recorded during the CRS tests and the loading force was measured with a pressure transducer. The procedure was repeated until all the samples were submitted to the CRS tests.

## 3. Results

### 3.1. Hydromechanical Properties of Compacted Loess

In this section, the compression curves after calibration are presented here and the hydromechanical parameters are derived. Meanwhile, the variation trend of these data were analyzed and discussed.

**Figure 4:**
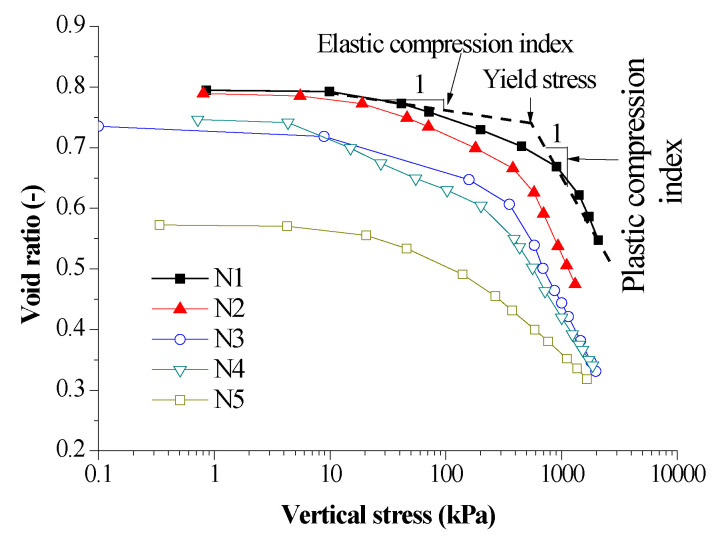
Curves of vertical stress—void ratio.

As shown in Figure 4, the compression curves of stress—void ratio data points were obtained from CRS tests and the number of data points could be collected according to the accuracy requirements. In addition, the void ratio decreased with the increase of vertical stress. When the initial vertical stress was small, the void ratio was large. When the vertical stress was higher than a certain value, the compression deformation increased. The deformation curves exhibit a bilinear shape, which was typical compression curves of reconstituted soil. According to the shape of compression deformation curves, the curve could be divided into elastic compression and plastic compression phase. The deformation of elastic phase was small, which was mainly the elastic deformation of soil skeleton. When the compression curve of the compressed loess was located in the plastic compression phase, the plastic deformation of the compacted loess skeleton occurs and was partially irreversible compression deformation of the sample after unloading. At this stage, the gas in the soil were partly discharged from the soil sample, the arrangement of soil particles will change, and the pore in the soil were smaller after being compressed.

**Figure 5:**
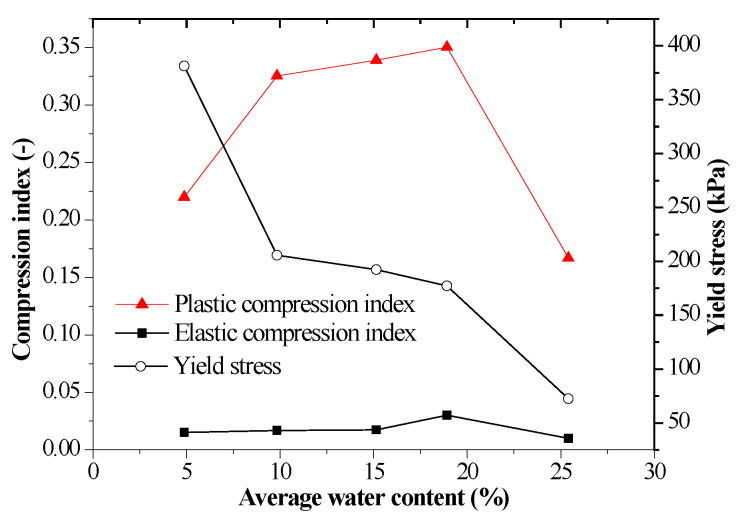
Evolution of mechanical parameters—average water content.

Figure 5 shows evolution of mechanical parameters versus average water content. The parameters can be determined as illustrated in Figure 4, which has been reported in [13,14]. Elastic compression index is an elastic stiffness parameter for changes in vertical stress for elastic states of the soil and plastic compression index is a stiffness parameter for changes in vertical stress for virgin states of the soil. Yield stress is the vertical stress that corresponds point of intersection of two lines as plotted in Figure 4. It was observed that the elastic compression index and plastic compression index increase with the increase of average water content except for those with the maximum average water content, and the yield stress decreases with the increase of average water content. The average water content was the average value of initial and final water content. Similar studies [36,37,38] also found that the increase of water content makes the compressibility of soil increase, which indicates that the soil softens. The phenomena could be explained from two aspects: one was that as the water content of soil increases, the water membrane between particles thickens so that particles tend to slide and pores collapse as force was applied on the soil, which will give rise to greater deformation; The other was that existence of water softens particles themselves, water makes the cohesive particles in soil expand though the proportion of clay minerals in loess was small. The expanded particles had more compressibility and reduce their strength. Moreover, dissolution of some salt minerals after encountering water also resulted in the decrease of sample strength. Hence, water plays a major role in softening soil samples and the softening effect was more obvious with the increase of water content. In addition, it must be noticed that the compression index of the soil decreased for the sample with a maximum average water content. This could be attributed to the water sensitivity of loess. As the loess suffers from water submersion, large deformation occurs under the gravity of the soil or a pressure larger than a given value. As illustrated in Figure 4, the sample with a maximum average water content generated a small initial void ratio of 0.573 compared with the values of void ratio ranging from 0.795 to 0.735 for the rest of average water contents due to water sensitivity of the loess. The water sensitivity of the compacted loess sample increased initial deformation and decreased compression index, which is a reason that water submersion can be a technique to deal with metastable loessic subgrade for enhancing its stiffness.

### 3.2. Electrical Resistivity Characteristics of Compacted Loess

After measurement of sample impedance, the electrical resistivity can be obtained using Equation (1). The variation trend of vertical and horizontal electrical resistivity is analyzed and discussed here.

**Figure 6:**
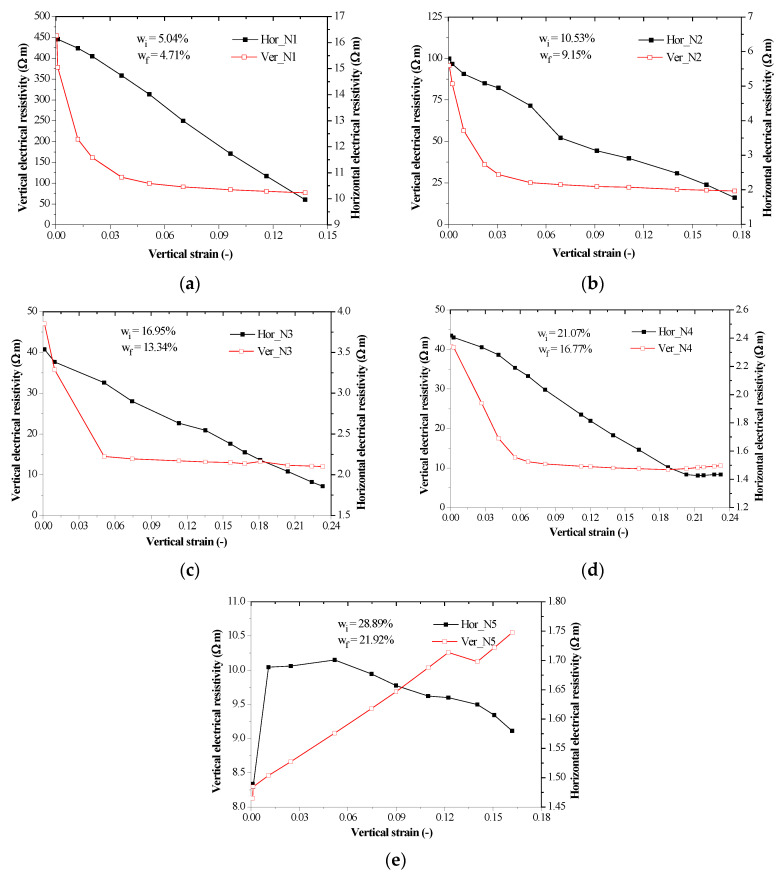
Horizontal and vertical electrical resistivity versus vertical strain of compacted loess samples with different initial and final water contents ranging from 5% to 28% under a current frequency of 50 kHz: (**a**) N1 sample, (**b**) N2 sample, (**c**) N3 sample, (**d**) N4 sample and (**e**) N5 sample.

The relationship between electrical resistivity and vertical strain at current frequency of 50 kHz is plotted in Figure 6. Owing to stability of impedance measurement of samples, the current frequency of 50 kHz was selected herein. The resistivity was divided into vertical resistivity and horizontal resistivity. It observes that the vertical electrical resistivity determined by a pair of electrodes installed at the top and bottom end of the sample was larger than the horizontal electrical resistivity obtained by a pair of electrodes at the radial lateral sides of the sample. This indicated that the vertical microscopic structure of the sample was different from the horizontal microscopic structure of the sample. In other words, the soil exhibits anisotropy. Moreover, compared with Figure 6a–e, it could be seen that both of the vertical and horizontal electrical resistivity decrease as water content increased. Similar observations have been derived by several studies [39,40,41,42]. This shows that water had an insignificant impact on the electrical resistivity, which may be attributed to be that water itself had a lower electrical resistivity compared with air in an unsaturated sample and the sample with a higher water content gets more water filling the pores of the sample rather than air. In other words, a higher soil saturation caused a lower electrical resistivity of the soil containing water with a given electrical resistivity. In addition, it observes that the vertical electrical resistivity decreased as vertical strain increased, but it had a different trend for the N5 sample with a maximum water content. Nonetheless, the horizontal electrical resistivity decreased as vertical strain increased. This indicated that compression increased the vertical electrical resistivity of the unsaturated compacted soil because the pores become small and air was squeezed out of the soil and saturation increased, whereas compression increased the vertical electrical resistivity of the saturated soil because water was squeezed out of the sample and soil particles that had a lower electrical resistivity compared with water progressively plays a major role to affect the electrical resistivity of the sample. In comparison of the vertical electrical resistivity and horizontal resistivity of N5 sample, the horizontal electrical resistivity was slightly decrease as the vertical strain increased, since the saturated sample compression had a less impact on the horizontal electrical resistivity. For N1–N4 samples with a water content from 5% to 20%, the relationship curves of vertical electrical resistivity versus vertical strain can be divided into two phases; the first phase had a quick decrease—this may be due to sample particle rearrangement or contact issue between electrodes and sample [40]. The second phase had a mild decrease due to increasing saturation and soil particle rearrangement.

In addition, the resistivity can be used to characterize the structural properties of soil. First, Archie [15] proposed the formation factor F of soil and characterized its structure with the resistivity. Since then, many scholars have proposed the concept of other resistivity parameters related to soil structure, including formation factor F, anisotropy index A and shape factor f. Among them, the formation factor F is a comprehensive reflection of the microstructure characteristics of the soil, which is the ratio of the soil resistivity to the pore water resistivity. For anisotropic soil, the formation factor can be divided into vertical formation factor F_V_ and horizontal formation factor F_H_ in terms of different directions and the equations are shown in Equation (2). The average formation factor $\bar{F}$ of soil is shown in Equation (3). Moreover, the anisotropy index A quantitatively reflects the anisotropy of soil as shown in Equation (4). Finally, the average shape factor $\bar{f}$ is the parameter of soil particle shape as shown in Equation (5). More details can be found in [17].

(2) $$F_{V}=\rho_{V}/\rho_{W}\cdot F_{H}=\rho_{H}/\rho_{W}$$

(3) $$\bar{F}=\left(F_{V}+2F_{H}\right)/3$$

(4) $$A=\sqrt{F_{V}/F_{H}}$$

(5) $$\bar{F}=n^{-\bar{f}}$$

where, ρ_W_ is the resistivity of pore water (Ω·m); ρ_V_ is the vertical resistivity of soil (Ω·m); ρ_H_ is the horizontal resistivity of soil (Ω·m); $\bar{f}$ is the average shape factor (dimensionless); n is the porosity (%).

According to the vertical and horizontal resistivity values shown in Figure 6 and the measured water resistivity of 50 KHz is 2.973 Ω·m, the average formation factor, anisotropy index and average shape factor of samples can be calculated in the light of Equations (3)–(5). Vertical strain is the macroscopic manifestation of soil sample due to structure change in the process of compression of compacted loess, the relationship between electrical parameters and vertical strain is thus established to characterize the relationship between microstructure and macroscopic deformation. The results are analyzed as follows:

#### 3.2.1. Average Formation Factor

After acquisition of electrical resistivity of samples, the average formation factor can be obtained using Equation (3). The variation trend of average formation factor is analyzed and discussed here.

**Figure 7:**
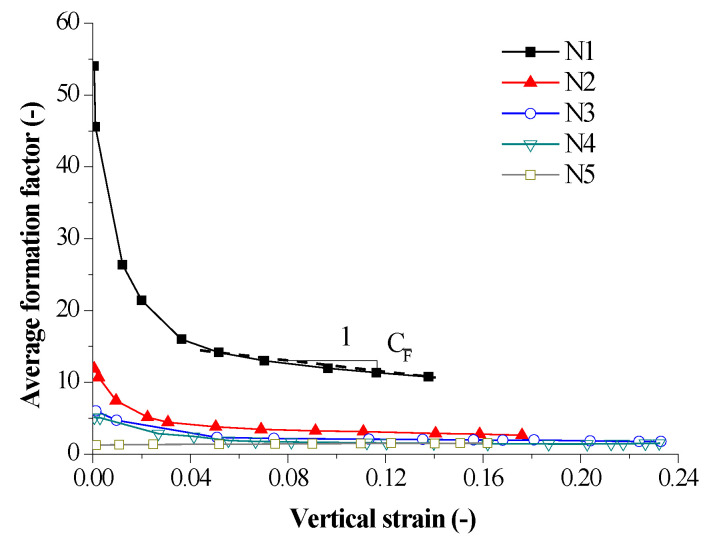
Relationship between average formation factor and vertical strain.

The relationship between the average formation factor and vertical strain is shown in Figure 7. Soil compression deformation can be attributed to vertical and horizontal structural changes due to the rearrangement of soil structure and the formation of new structure. It was observed that the average formation factor decreases with the increase of vertical strain except for the N5 sample, which was similar to variation trend of vertical resistivity and indicated that the dominant role of vertical formation factor. The decrease of average formation factor was mainly caused by the decrease of porosity and the increase of saturation. According to [43], when the soil was compacted, the inter-aggregate pores in the soil were reduced and the intra-aggregate pores were unchanged. The explanation of average formation factor reduction with an increase of vertical strain could be the increase of sample saturation as vertical strain increases. In a double linear coordinate, the average formation factor almost bilinearly decreases as vertical strain increases except for the sample with the maximum water content. This indicated that the soil structure changes, and sample saturation increases with the continuous compression. Since the change of vertical strain was mainly attributed to the change of macroscopic pores in the soil, the average formation factor could reflect the stability of compacted loess. In addition, it was noticed that at a higher water content, such as the N5 curve and the end of the N4 curve, the average formation factor increases as the vertical strain decreases, which indicated that the sample was saturated, and water was discharged from the sample. For a given vertical strain, the structural factor decreased as water content increased. This indicates that water content had an important impact on the average formation factor. Therefore, the stability of compacted loess in the practical project can be evaluated by taking into account using the formation factor to characterize the vertical strain by the linear relationship.

#### 3.2.2. Anisotropy Index

After acquisition of electrical resistivity of samples, the anisotropy index can be obtained using Equation (4). The variation trend of anisotropy index is analyzed and discussed here.

**Figure 8:**
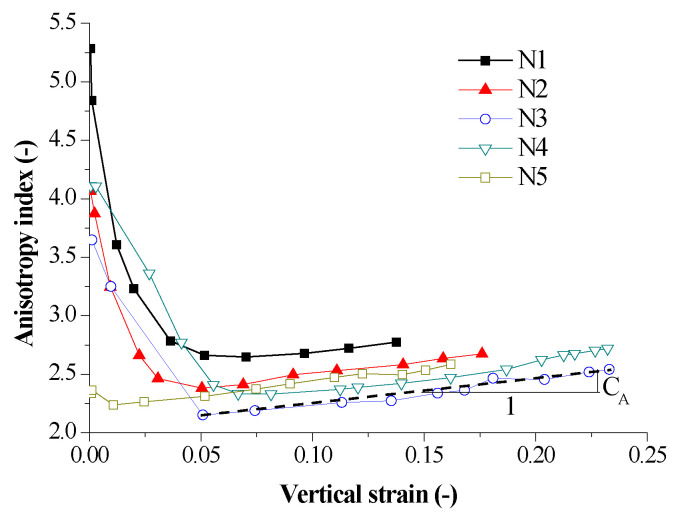
Relationship between anisotropy index and vertical strain.

Due to the difference of the vertical and the horizontal structure, soil including compacted loess exhibits anisotropy. The anisotropy index calculated from the vertical and horizontal formation factors obtained from the soil resistivity can reflect the anisotropy of the soil. The relationship between the anisotropy index and vertical strain during the compression of compacted loess is shown in Figure 8. It was observed that in the initial compression stage, the anisotropy index of soil decreases significantly with the increase of vertical strain; In the second compression stage, the anisotropy index tends to slightly increase. This may be because a structural anisotropy with a preferential orientation of the particles towards the horizontal for the remolded samples in the initial state and the samples undergo particle reorientation during loading [44,45]. The change rate of soil’s horizontal structure was larger than that of soil’s vertical structure. In the light of [46,47], the coefficient of at-rest lateral pressure coefficient increases with the increase of stress and was less than 1, which could reflect variation of anisotropy of soil. According to the ratio of vertical and horizontal resistivity, it seems that the anisotropy index was reciprocal with the at-rest lateral pressure coefficient. In addition, there was difference between the anisotropy index and the lateral static pressure coefficient because the anisotropy index initially decreases and then increases, whereas the lateral static pressure coefficient usually increases then tend to a stable state as vertical strain increases. Moreover, it seems that the anisotropy index at the second stage initially decreased then increased as water content increased. The minimum anisotropy index of compacted sample had a water content of about 15.145%, which was close to the optimum moisture content of 16.7%. It seems that the variation of anisotropy index with water content here was reverse to variation of dry density with water content by proctor compaction test. In other words, the sample with an optimum moisture content had a minimum anisotropic property. Higher or lower water content of compacted sample generated higher anisotropy indices and samples with a water content at the left side of optimum exhibits higher anisotropy index than those at the right side of optimum.

#### 3.2.3. Average Shape Factor

After acquisition of electrical resistivity of samples, the average shape factor can be obtained using Equation (5). The variation trend of average shape factor is analyzed and discussed here.

**Figure 9:**
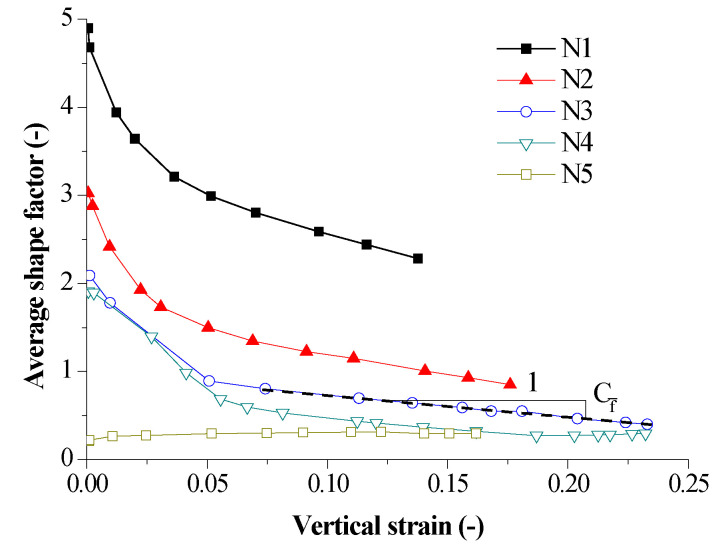
Relationship between average shape factor and vertical strain.

The compression process of compacted loess was accompanied by the deformation of soil structural elements, such as the rearrangement of soil particles and the change of cementation between soil particles. The relationship between the average shape factor and the vertical strain during the compression of compacted loess was shown in Figure 9. It observes that in the first compression stage, the average shape factor sharply decreases with the increase of vertical strain, followed by a slow decrease as the vertical strain increases in the second compression stage, which basically showed a double linear shape. In the context of [48,49], loess cementitious materials mainly include calcium, clay minerals, organic colloids, soluble salts, etc., which had three types of distribution formations in the compacted loess [50]. With the increase of pore water in the compressed loess, dissolution and softening of cementitious materials make the cementation between soil particles decrease [51]. Meanwhile, loess compression leads to structural damage of soil, rotation or sliding of soil particles, the degree of cementation between soil particles thus continues to weaken. All of these factors result in the decrease of the average shape factor. In addition, it must notice that the shape factor of N5 sample increased as vertical strain increased, which indicated that as water was discharged from the saturated compacted loess, the particle cementation increased. The change trend of cementation between soil particles in the process of soil compression could be quantitatively analyzed by using the average shape factor.

#### 3.2.4. Relationship between Electrical Parameters and Hydromechanical Parameters

In order to quantitatively analyze the hydromechanical properties of compacted soil and evaluate the stability of loessic ground or subgrade, it was of importance to establish the relevance between the electrical parameters computed from the directly measured electrical impendence of samples and their hydromechanical parameters including vertical strain and water content and so forth. In terms of the curves plotted in Figure 7, Figure 8 and Figure 9, the electrical parameters were quantitively correlated with soil hydromechanical parameters.

In order to accurately depict the curves, different coefficients were defined. The coefficient of average formation factor $C_{\bar{F}}$ can be defined as $C_{\bar{F}}=-\Delta \bar{F}/\Delta \varepsilon$. The coefficient of anisotropy index $C_{A}$ can be defined as $C_{A}=\Delta A/\Delta \varepsilon$ and the coefficient of average shape factor $C_{\bar{f}}$ can be defined as $C_{A}=-\Delta \bar{f}/\Delta \varepsilon$. There, Δ is the symbol that is representative of increment of a variable.

**Figure 10:**
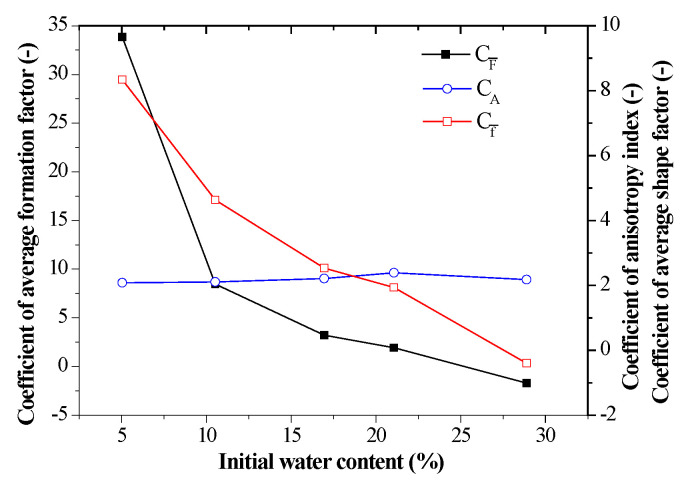
Relationship between structural coefficients and initial water content.

Figure 10 shows the relationship between electrical coefficients and initial water content. It observed that the coefficients of average formation factor and average shape factor decrease with an increase of initial water content, while there was less variation of the coefficients of anisotropy index and had an average value of 2.190.

For the sake to evaluate the hydromechanical properties of in situ loess ground or subgrade by the resistivity method, it is necessary to establish the relationship between structural parameters and soil hydromechanical parameters. In this work, the relationship between average formation factor and both of initial water content and vertical strain is derived to predict the hydromechanical properties by measuring the electrical impedance of the soil in the field. Taking into account for the soil undergoing a strain larger than a given value (about 0.04) is used to fit the relevance that is shown in Equation (6):

(6) $$\bar{F}=C_{\bar{F}}\varepsilon +b$$

where, b is the intercept of $\bar{F}$ as vertical strain ε = 0. The intercept b is assumed to be a function of initial water content.

In Figure 9, the correlation of coefficients of average formation factor and initial water content can be regressed by a polynomial. It also observes that the intercept b varies with water content and their relationship can also be fitted by a polynomial. Hence, the Equation (6) can be regressed to Equation (7):

(7) $$\bar{F}=\left(-0.007w_{i}^{3}+0.466w_{i}^{2}-9.9w_{i}+71.745\right)\varepsilon -0.002w_{i}^{3}+0.121w_{i}^{2}-2.956w_{i}+25.299$$

where, w_i_ is the initial water content (%).

The Equation (7) can be used to compute the vertical strain after obtaining the average formation factor from measurement of soil impedance. This can provide a convenient means to obtain the hydromechanical parameters of soil ground and subgrade, such as strain, void ratio etc. Meanwhile, the rest of electrical parameters can also be correlated with the hydromechanical parameters of the compacted loess as mentioned above, which can form a comprehensive indicators to characterize the hydromechanical properties of compacted soil in the field and laboratory.

## 4. Conclusions

In this work, an electrical resistivity method of characterizing hydromechanical and structural properties of compacted loess during constant rate of strain compression was studied. The electrical parameters, including the average formation factor, the anisotropy index and the average shape factor, are determined by the electrical resistivity method. The relationship between the hydromechanical and structural properties of compacted loess and these electrical parameters is qualitatively and quantitatively analyzed and discussed to explore the application of the resistivity method of characterizing the hydromechanical and structural properties of compacted loess in the field and laboratory. The main conclusions are obtained as follows:

The CRS method could quickly obtain continuous compression curve points. The compression curves could be divided into elastic compression stage and plastic compression stage. The plastic indices increased from 0.220 to 0.350 and the elastic indices increased from 0.0152 to 0.030 with an increase of average water content ranging from 4.88% to 18.92%, but they decreased to 0.167 and 0.010 at the maximum average water content of 25.40%, while yield stress decreased from 381.28 kPa to 72.35 kPa with the increase of average water content. The plastic compression index was ten times greater than the elastic compression index. In addition, the sample with a maximum average water content generated the smallest initial void ratio of 0.573 than the rest of values of initial void ratio ranging from 0.795 to 0.735, which indicated the water sensitivity of the compacted loess.

According to the relationship between vertical resistivity and vertical strain of compacted loess, the soil compression curves can be divided into two stages. In the first stage, the vertical resistivity rapidly decreased, while it mildly decreased in the second stage. The vertical resistivity of compacted loess with lower water content decreases with the increase of vertical strain, but that with the maximum water content increases with the increase of vertical strain. The horizontal resistivity of compacted samples decreases with the increase of vertical strain. Moreover, both of horizontal and vertical resistivity decreases with an increase of water content. The initial values of horizontal and vertical resistivity range from 16.249 to 1.489 and from 453.806 to 8.129 as water content increases, respectively. The final values of horizontal and vertical resistivity range from 9.964 to 1.580 and from 76.685 to 10.549 as water content increases, respectively.

In addition, the average formation factor and the average shape factor of N1–N4 samples decreased with the increase of vertical strain, whereas those of N5 sample increased with the increase of vertical strain. The average formation factor and the average shape factor decreased as water content increased. The initial values of average formation factor and the average shape factor varied from 54.034 to 1.234 and from 4.897 to 0.208 as water content increased, respectively. The final values of average formation factor and the average shape factor varied from 10.735 to 1.523 and from 2.284 to 0.296 as water content increases, respectively. This indicated that decrease of the average formation factor and the average shape factor could improve the stability of soil mass as water content was low but increases of the average formation factor and the average shape factor could improve the stability of soil mass with a high water content. The limit between the low and high water content was about 20% and needs to be further determined by using samples with smaller water content interval. In addition, the anisotropy index first decreased and then tended to slightly increase with the increase of vertical strain. The anisotropy index first decreased and then increased with an increase of water content. The initial and final values of anisotropy index ranged from 5.285 to 2.336 and from 2.744 to 2.584 as water content increased, respectively. It seems that the anisotropy index was related to at-rest lateral pressure coefficient and optimum dry density. In sum, water content and vertical strain had a significant impact on the average formation factor, the average shape factor and the anisotropy index, which indicated water content and vertical strain had a significant impact on the microscopic manifestation of sample. The structural properties of compacted loess in the process of compression could be characterized by the variation of these electrical parameters.

Moreover, the relationship between the electrical parameters and the hydromechanical parameters is quantitatively analyzed. Coefficients of average formation factor, anisotropy index and average shape factor are defined as functions of initial water content. The coefficients of average formation factor decrease from 33.830 to −1.698 and the coefficients of average shape factor decreased from 8.339 to −0.398 as water content increased, whereas there was less variation for the coefficient of anisotropic index with a value of 2.190. An equation correlating average formation factor and water content and vertical strain was regressed to characterize the hydromechanical properties of compacted loess samples by measuring their impedance.

Electrical resistivity method can be used to monitor and evaluate the stability of loessic ground and subgrade. For example, the electrodes can be placed in the soil to obtain the electrical indices during treatment and access the compaction effectiveness by using the electrical indices as indicators. Moreover, this method can be used to determine the deformation or density of samples in the laboratory. Despite good application prospects, it further study is still needed to improve this method and technique, such as taking into account for the influence of the water mineral composition and other factors, etc.

## Figures and Tables

**Table 1:** Basic physical properties of the soil.

Specific Gravity	Maximum Dry Density	Optimum Moisture Content	Liquid Limit	Plastic Limit	Plasticity Index	Coefficient of Collapsibility
**G**_**s**_ **(−)**	**ρ**_**max**_**(g·cm**^**−3**^)	**w**_**opt**_ **(%)**	**w**_**L**_ **(%)**	**w**_**P**_ **(%)**	**I**_**p**_ **(−)**	**Δs (−)**
2.7	1.77	16.7	25.5	16.9	8.6	0.019

**Table 2:** Parameters of the compacted loess soil.

Sample	Initial Height	Initial Dry Density	Initial Water Content	Final Water Content
	H_i_ (mm)	ρ_d_ (g·cm^−3^)	w_i_ (%)	w_f_ (%)
N1	10.30	1.504	5.04	4.71
N2	10.27	1.509	10.53	9.15
N3	9.96	1.556	16.95	13.34
N4	10.03	1.546	21.07	16.77
N5	9.03	1.717	28.89	21.92
